# Supplementary material for: Molecular dynamics-based refinement and validation for sub-5 Å cryo-electron microscopy maps
Source: eLife. 2016 Jul 7;5:e16105. doi: 10.7554/eLife.16105 (PMC4990421; doi:10.7554/eLife.16105)
Supplement: Supplementary file 1. — (A) TRPV1 MDFF Results. (B) Structure quality indicators for TRPV1 structures. (C) MDFF for the TRPV1 TM region. (D) Structure quality indicators for γ-secretase. (E) Measures of fit for MDFF refinements of β-galactosidase prepared initially at 1000 K. (F) Structural quality indicators for MDFF-refined β-galactosidase prepared initially at 1000 K. DOI: http://dx.doi.org/10.7554/eLife.16105.028 [file elife-16105-supp1.pdf]

Molecular dynamics-based model refinement and validation with Resolution Exchange MDFF for sub-5 Å cryo-electron microscopy maps: Supplementary file 1

Abhishek Singharoy[†,1], Ivan Teo[†,2,1], Ryan McGreevy[†,1],

John E. Stone[1], Jianhua Zhao[3], Klaus Schulten[2,1,**]

[1] Beckman Institute for Advanced Science and Technology, University of Illinois at Urbana-Champaign

[2] Department of Physics, University of Illinois at Urbana-Champaign, Urbana, IL 61801, USA

[3] Department of Biochemistry and Biophysics, University of California San Francisco School of Medicine

[†] These authors contributed equally to this work.

** Corresponding author

Email: kschulte@ks.uiuc.edu

Tel: 1-217-244-1604

Supplementary file 1A: TRPV1 MDFF Results. Similar to the results for β-galactosidase (Table 1), cMDFF and ReMDFF refinement of TRPV1 produce the best fitted models as analysed by various metrics. The models obtained through both cMDFF and ReMDFF are better or equal to the *de novo* structure in every analysis. Numbers in parentheses are representative of the whole structure (tetramer) while the main entries refer only to the single monomer that was fit.

Structure	RMSD	EMRinger	iFSC1	iFSC2	MolProb.	GCC
de novo	0.0	0.83	2.33 (3.48)	2.62 (4.33)	3.92	0.53 (0.72)
Refined *de novo*	1.1	1.75	1.90 (3.52)	2.13 (4.42)	1.34	0.54 (0.73)
Initial	24.1	0.62	0.67 (2.96)	0.77 (3.81)	3.91	0.16 (0.67)
Direct MDFF	7.9	1.51	1.61 (3.68)	1.79 (4.47)	1.32	0.50 (0.72)
cMDFF	2.4	1.68	2.37 (3.66)	2.62 (4.43)	1.36	0.54 (0.72)
ReMDFF	2.5	1.99	2.41 (3.68)	2.75 (4.50)	1.47	0.53 (0.73)

Supplementary file 1B: Structure quality indicators for TRPV1 structures. TRPV1 structures investigated in the present study were uploaded to the MolProbity server (`http://molprobity.biochem.duke.edu`) to extract the quantities presented below. As in the case of β-galactosidase, the overall MolProbity score has been improved, relative to the *de novo* and initial structures, by cMDFF and ReMDFF at the expense of a small increase in Ramachandran outliers and Cβ deviations.

	de novo (?)	Refined *de novo*	Initial	Direct MDFF	cMDFF	ReMDFF
Clashscore	92.8	0.0	91.6	0.0	0.0	0.0
Poor rotamers (%)	28.8	3.1	28.8	2.4	2.8	4.4
Favored rotamers (%)	53.8	90.5	53.8	92.8	91.4	87.6
Ramachandran outliers (%)	1.0	3.4	1.0	3.5	3.5	3.4
Ramachandran favored (%)	94.5	92.3	94.5	90.8	91.1	92.3
MolProbity	3.92	1.34	3.91	1.32	1.36	1.47
Cβ deviations (%)	0.0	0.53	0.0	0.26	0.32	1.01
Bad bonds (%)	0.72	0.0	0.77	0.0	0.0	0.0
Bad angles (%)	0.52	0.42	0.51	0.41	0.37	0.50
RMS distance (Å)	0.019 (0%)	0.019 (0%)	0.017 (0%)	0.019 (0%)	0.019 (0%)	0.019 (0%)
RMS angle (degrees)	1.9 (0.018)	1.9 (0.018%)	2.0 (0.240%)	1.9 (0.014%)	1.9 (0.014%)	1.9 (0.032%)
Cis prolines (%)	15.38	15.38	15.38	15.38	15.38	15.38
Cis non-prolines (%)	0.62	0.62	0.62	0.62	0.62	0.62

Supplementary file 1C: MDFF for the TRPV1 TM region. Three MDFF refinements of the TRPV1 TM region were performed under different conditions. Measures of quality of the resulting structures are compared with those for the published structure and for the structure obtained from the Rosetta software. The tabulated results show that MDFF with backbone atoms free to move fared as well or better than Rosetta in terms of the measures considered.

Structure	RMSD	EMRinger	iFSC1	iFSC2	MolProb.	GCC
de novo	0.0	1.05	3.20	4.28	4.02	0.63
Rosetta	1.2	2.57	3.55	4.60	1.55	0.63
Backbone restrained	1.8	2.34	3.59	4.71	1.08	0.63
Backbone free	1.2	2.51	3.80	4.85	1.37	0.64

Supplementary file 1D: Structure quality indicators for γ-secretase. Local structural statistics for the reported and refined structures of γ-secretase are presented below.

	de novo (**?**)	Refined *de novo*
Clashscore	52.1	0.0
Poor rotamers (%)	13.8	2.0
Favored rotamers (%)	74.8	93.0
Ramachandran outliers (%)	2.0	1.3
Ramachandran favored (%)	91.6	93.0
MolProbity	3.56	1.17
Cβ deviations (%)	0.70	1.39
Bad bonds (%)	0.33	0.0
Bad angles (%)	0.20	0.33
Cis prolines (%)	0.0	0.0
Cis non-prolines (%)	0.0	0.0

Supplementary file 1E: Measures of fit for MDFF refinements of β-galactosidase prepared initially at 1000 K.

Structure	RMSD	EMRinger	iFSC1	iFSC2	MolProb.	GCC
de novo (**?**)	0.0	2.25	4.03	5.00	3.14	0.67
Initial	7.6	0.26	0.10	0.09	2.22	0.25
Direct MDFF	6.2	1.91	2.11	2.73	1.88	0.47
cMDFF	3.2	2.88	3.17	3.96	1.74	0.63
ReMDFF	3.0	2.89	3.34	4.15	1.84	0.64

Supplementary file 1F: Structural quality indicators for MDFF-refined β-galactosidase prepared initially at 1000 K.

	de novo (?)	Initial	Direct MDFF	cMDFF	ReMDFF
Clashscore	53.7	0.0	0.0	0.0	0.0
Poor rotamers (%)	11.6	24.8	7.6	5.4	6.7
Favored rotamers (%)	67.4	53.7	81.4	85.0	85.4
Ramachandran outliers (%)	0.2	5.0	8.7	7.8	8.5
Ramachandran favored (%)	97.4	84.7	81.9	83.4	81.6
MolProbity	3.14	2.22	1.88	1.74	1.84
Cβ deviations (%)	0.0	20.6	0.8	0.7	3.7
Bad bonds (%)	0.09	15.4	0.03	0.02	0.02
Bad angles (%)	0.03	18.71	1.38	1.20	1.40
Cis prolines (%)	8.06	8.06	8.06	8.06	8.06
Cis non-prolines (%)	1.15	1.15	1.15	1.15	1.15
