# Supplementary material for: Molecular dynamics-based refinement and validation for sub-5 Å cryo-electron microscopy maps
Source: eLife. 2016 Jul 7;5:e16105. doi: 10.7554/eLife.16105 (PMC4990421; doi:10.7554/eLife.16105)
Supplement: Supplementary file 2. — DOI: http://dx.doi.org/10.7554/eLife.16105.029 [file elife-16105-supp2.docx]

PDB structure files of β-galactosidase for

1. Refined de novo - 3J7H-refdenovo.pdb

2. Initial - 3J7H-initial.pdb

3. Direct MDFF - 3J7H-direct.pdb

4. cMDFF - 3J7H-cMDFF.pdb

5. ReMDFF - 3J7H-ReMDFF.pdb

Figure 3 source data

1. PDB file containing 12 different structures of β-galactosidase sampled from the MD simulationtrajectory from which local RMSFs were obtained - 3J7H-ensemble.pdb

2. PDB file containing structure of β-galactosidase with beta field set to local RMSF squared values - 3J7H-RMSF2.pdb

3. PDB file containing structure of TRPV1 with beta field set to local RMSF squared values - 3J5P-RMSF2.pdb

PDB structure files of TRPV1 for

1. Refined de novo - 3J5P-refdenovo.pdb

2. Initial - 3J5P-initial.pdb

3. Direct MDFF - 3J5P-direct.pdb

4. cMDFF - 3J5P-cMDFF.pdb

5. ReMDFF - 3J5P-ReMDFF.pdb

PDB structure files of TRPV1 transmembrane domain for

1. PDB structure refined with backbone restraints - 3J5P-TM-bbres.pdb

2. PDB structure refined without backbone restraints - 3J5P-TM-bbfree.pdb

PDB structure files of β-galactosidase with low-quality search model refinements, for

1. Refined de novo - 3J7H-1000K-refdenovo.pdb

2. Initial - 3J7H-1000K-initial.pdb

3. Direct MDFF - 3J7H-1000K-direct.pdb

4. cMDFF - 3J7H-1000K-cMDFF.pdb

5. ReMDFF - 3J7H-1000K-ReMDFF.pdb
